# Use of the Bacterial Lysate OM-85 in the Paediatric Population in Italy: A Retrospective Cohort Study

**DOI:** 10.3390/ijerph18136871

**Published:** 2021-06-26

**Authors:** Anna Cantarutti, Elisa Barbieri, Antonio Scamarcia, Luigi Cantarutti, Cristina Canova, Carlo Giaquinto

**Affiliations:** 1 Unit of Biostatistics, Epidemiology and Public Health, Department of Statistics and Quantitative Methods, University of Milano-Bicocca, 20126 Milan, Italy; anna.cantarutti@unimib.it; 2 National Centre for Healthcare Research and Pharmacoepidemiology, Department of Statistics and Quantitative Methods, University of Milano-Bicocca, 20126 Milan, Italy; 3 Società Servizi Telematici-Pedianet, 35121 Padua, Italy; a.scamarcia@sosepe.com (A.S.); l.cantarutti@sosepe.com (L.C.); carlo.giaquinto@unipd.it (C.G.); 4 Division of Pediatric Infectious Diseases, Department for Woman and Child Health, University of Padua, 35128 Padua, Italy; 5 Unit of Biostatistics, Epidemiology and Public Health, Department of Cardiac, Thoracic, Vascular Sciences and Public Health, University of Padova, 35128 Padova, Italy; cristina.canova@unipd.it

**Keywords:** OM-85, respiratory tract infections, paediatric primary care

## Abstract

Background: In Italy, the bacterial lysate OM-85 (Broncho-Vaxom^®^, Broncho-Munal^®^, Ommunal^®^, Paxoral^®^, Vaxoral^®^) is registered for the prophylaxis of recurrent respiratory tract infections (RTIs) in adults and children above one year of age, but there are limited data on its use in the paediatric population. We aim to estimate the impact of OM-85 treatment on RTIs and antibiotic prescriptions in children. Methods: This study included children aged 1 to 14 years enrolled in Pedianet, a paediatric general practice research database, from January 2007 to June 2017, having at least one prescription of OM-85. Children with less than 12 months of follow-up before (PRE period) and after (POST period) the OM-85 prescription were excluded. The frequency of antibiotic prescriptions and the frequency of RTI episodes in the PRE and POST periods were compared through the post-hoc test. Subgroup analysis was performed in children with recurrent RTIs. Results: 1091 children received 1382 OM-85 prescriptions for a total follow-up of 619,525.5 person-years. Overall, antibiotic prescriptions decreased from a mean of 2.8 (SD (standard deviation) 2.7) prescriptions in the PRE period to a mean of 2.2 (SD 2.6) prescriptions in the POST period (*p* < 0.0001). RTIs decreased from a mean of 3.4 (SD 2.9) episodes in the PRE period to a mean of 2.5 (SD 2.6) episodes in the POST period (*p* < 0.0001). No change in antibiotic class was noted, and co-amoxiclav remained the preferred therapy in 28% of cases, followed by amoxicillin. These results were confirmed among children with recurrent RTIs. Conclusions: OM-85 is effective in preventing both antibiotic prescriptions and RTIs in children.

## 1. Background

Recurrent respiratory tract infections (RTIs) are one of the most common conditions affecting paediatric patients, with a significant impact both in terms of morbidity, antibiotic usage, reduction in quality of life, as well as an increase in healthcare utilisation [1].

In the past decades, different studies investigating bacterial lysates, such as OM-85 (Broncho-Vaxom^®^, Broncho-Munal^®^, Ommunal^®^, Paxoral^®^, Vaxoral^®^), have shown their efficacy in the prevention of recurrent RTIs in children when taken orally, showing a decreased risk of respiratory tract infection ranging from 16% to 40%, with an excellent tolerability profile [2,3,4]. OM-85 is a vaccine constituted by 21 strains of 8 different bacteria, most commonly involved in RTIs (including Haemophilus influenzae, Streptococcus pneumoniae, Klebsiella pneumoniae ssp. pneumoniae, ssp. ozaenae, Staphylococcus aureus, Streptococcus pyogenes and sanguinis, and Moraxella catarrhalis).

The mechanism of action is not completely clear, as well as the role of OM-85 in stimulating humoral and cellular immunity. Pre-clinical data indicate that OM-85 stimulates the innate and adaptive immune response through the activation of dendritic cells, an increase in the Th1 response, and an increase in secretory and serum IgA [4,5,6,7]. In particular, OM-85 has the ability to induce interferon-β production, an important cytokine with immunomodulatory and antiviral effects [8]. It has also been shown that it keeps innate immune cells in a pre-activated state, releasing large amounts of the inflammasome IL-1 upon sensing an inflammasome trigger, and potentially reducing selected viral infections [7].

In Italy, OM-85 is registered in adults and children above one year of age for the prophylaxis of recurrent RTIs [9]. Few studies showed its efficacy in preventing bacterial upper RTIs in children, and a study demonstrated that the combination of OM-85 with flu vaccine significantly reduced the incidence of RTIs in children < 6 years of age [4,5,10]. However, the high heterogeneity of the studies evaluating the effect of the OM-85 and the poor to moderate quality of trials highlights discrepancies in the findings [11,12]. de Souza et al. did not report a significant difference in the number of RTIs between the treated and untreated group with OM-85 (*p* = 0.26). However, other studies found a significant relative reduction rate of RTIs, ranging from 40% to 43%. More information is needed on OM-85’s real-world use and long-term effectiveness.

The general practitioner and the family paediatrician (FP) are generally responsible for caring for children with RTIs, and only a few patients are referred to the hospital

We conducted a paediatric population-based cohort study, to evaluate the impact of OM-85 treatment on RTIs’ frequency and antibiotic prescriptions in Italian children.

## 2. Methods

### 2.1. Data Source

Italy is one of few countries where a specific primary care system is devoted to children up to 14 years of age. Within the framework of the Italian National Healthcare System, every child is registered at birth and receives medical care, free of charge, from one of the approximately 7000 FPs who work for the Italian National Healthcare System.

Pedianet is an organised network of FPs collecting electronic patient records for epidemiological and clinical research purposes. Data generated during routine patient care are collected and handled anonymously, in compliance with the Italian regulations, and stored under a unique numerical identifier. The Pedianet database records demographic and healthcare use data, in particular: (i) the prescription file containing information on all medications and vaccinations prescribed by the assigned FP (identified by the Anatomical Therapeutic Chemical (ATC) code), (ii) the medical file containing diagnostic and clinical information (free text or coded using the 9th International Statistical Classification of Diseases and Related Health Problems system (ICD-9 CM) code), (iii) specialist appointments, diagnostic procedures, and hospital admissions, and (iv) anthropometric measurements.

This study was conducted following the Declaration of Helsinki and the Guidelines of Good Epidemiology Practices.

The study and the access to the database were approved by the Internal Scientific Committee of So.Se.Pe. Srl, the legal owner of Pedianet.

### 2.2. Study Design, Population, and Case Identification

In this retrospective cohort study, all children aged 1 to 14 years, registered from 1 January 2007 to 30 June 2017, with one of the FPs who are collaborating with the continuous Pedianet data collection, and with at least one prescription of OM-85 (ATC code J07AX**), were included. The first OM-85 prescription is defined as the index prescription.

RTI cases were defined as children having a visit claim with an upper or lower RTI identified with ICD9-CM codes (Supplementary Table S1) or a descriptive diagnosis in the free text fields. Free text fields were manually evaluated to exclude any false-positive cases by two investigators (A.S. and E.B.), and when not in agreement, a consensus was achieved with a third investigator (C.G.). 

An RTI episode was comprised of one or more primary care RTI-related visits. If additional visits with the same diagnosis were found less than 30 days after the first visit for any patient, they were considered as follow-up visits. A recurrent RTI case was defined as a child having more than 6 episodes of RTI in 12 months. 

Comorbidity was defined as the presence of a chronic complex condition (i.e., cystic fibrosis, diabetes mellitus, asthma, cardiovascular problems, celiac disease), immunodeficiency or immunosuppressive therapy, prematurity (less than 37 weeks’ gestation), Down′s syndrome, renal failure, craniofacial malformation, and congenital cardiac disease reported in the diagnosis fields or the exemption table.

To have an even follow-up in the period before and after the index prescription, we considered just those children with at least 12 months of follow-up before and after the index prescription. For each child included in the study, we evaluated two periods: (i) the PRE period, defined as the 12 months before the OM-85 index prescription, and (ii) the POST period, defined as the 12 months after the OM-85 index prescription.

### 2.3. Statistical Analysis

Descriptive statistics were provided for patients with at least one prescription of OM-85.

The outcomes of interest were the number of antibiotic prescriptions and the number of RTI episodes per child in the PRE and POST periods. Prescription distribution of antibiotics, as a whole and stratified by class (amoxicillin, co-amoxiclav, II-gen. cephalosporins, III-gen. cephalosporins, macrolides, clofoctol, thiamphenicol, and other antibiotics), and distribution of RTI episodes were compared between PRE and POST periods. Comparison of categorical independent variables was performed with the chi-square test, while continuous variables were compared with the post-hoc test after assessing for normality and equality of variance.

Subgroup and stratified analyses were conducted only for children with recurrent RTIs for a total of 211 children, and by sex. 

## 3. Results

In the study period, 1091 children received 1382 prescriptions of OM-85 for a total follow-up of 619,525.5 person-years, from 1 January 2007 to the 30 June 2017. Children had a mean age of 4.3 (SD 2.3) years at first prescription, with no difference in sex (female 47% vs. male 53%). Less than 3% (31/1091) of children exhibited comorbidity (Table 1). The most prevalent chronic conditions were cardiovascular and congenital health diseases and asthma (22.6% for both).

Eighty percent of the children (876/1091) received one OM-85 prescription in the study period, up to a maximum of eight prescriptions for one child (Supplementary Table S2). All the prescriptions were for formulations accounting for 30 single doses.

The frequency of antibiotic prescriptions in the PRE and POST periods is described in Table 2 for the overall prescriptions and in Table 3 stratified by the antibiotic group.

More than 20% of the children received five or more antibiotic prescriptions in the PRE period. On the contrary, in the POST period, 26% (284/1091) of the children received no prescription, and around 90% of them received less than five prescriptions (*p* < 0.0001). The expected mean antibiotic prescriptions difference between PRE and POST periods is approximately −0.53. 

The most prescribed antibiotic either before and after OM-85 index prescription was co-amoxiclav, accounting for 29% of prescriptions in the PRE period and for 28% of prescriptions in the POST period. The second most prescribed was amoxicillin (27% vs. 25% in the PRE vs. POST periods), and the third was macrolides (19% vs. 19% in the PRE vs. POST periods).

Moreover, in the PRE period, more than 28% (309/1091) of the children included in the study had at least 5 RTI episodes, while 3% of them (34/1091) had from 10 to 14 RTI episodes (Table 4). In the POST period, 20% (214/1091) of children had no RTI episodes, 23% (254/1091) had one RTI episode, and less than 12% had more than five RTI episodes. The expected mean RTI difference between PRE and POST periods is approximately −0.65 (*p* < 0.0001).

### Subgroup and Stratified Analysis

The subgroup analyses conducted in the 221 children with recurrent RTIs (>6 RTI episodes in a year) confirmed the main results. 

In particular, in the PRE period, 60% of the children included in the analyses had five or more prescriptions, whereas, in the POST period, 73% of the children recorded less than five prescriptions (*p* < 0.0001) (Supplementary Table S3). The expected mean antibiotic prescriptions difference between PRE and POST periods is approximately −1.2 (*p* < 0.0001). Co-amoxiclav remained the most prescribed antibiotic in the PRE and POST periods, accounting for around 27% of the prescriptions, followed by the amoxicillin and macrolides class (complete data are shown in Supplementary Table S4).

In the PRE period, RTI frequency rates varied from 6 (28%) to 17 (1.42%), whereas in the POST period, 73% of the children had five or less episodes of RTIs. In particular, 5% had no RTIs (*p* < 0.0001) (Supplementary Table S5). The expected mean RTI difference between PRE and POST period is approximately −1.6.

The stratified analysis by sex (Supplementary Tables S6 and S7) confirmed the main results both in males and females, with expected mean antibiotic prescriptions and RTI differences between PRE and POST periods higher in males than in females (−0.56 vs. −0.49 and −0.66 vs. −0.63 respectively, for antibiotic prescriptions and RTIs in males and females).

## 4. Discussion

From 2007 to 2017, 1091 children received at least one OM-85 prescription. After the OM-85 prescription, the overall mean of antibiotic prescriptions and the number of RTIs decreased significantly. No change in antibiotic class was noted, and co-amoxiclav remained the preferred therapy in 28% of cases. Results were also consistent in the subgroup analyses restricted to children with recurrent RTIs and among sex. 

OM-85 was mainly prescribed to children below five years of age, and this is not a surprise since the immaturity of children′s immune systems [13] is one of the leading causes of RTIs, and the presence of a concomitant comorbidity increases the frequency and the exacerbation of the infection [14,15]. In fact, the mechanism of action of vaccines, such as OM-85, is to stimulate the activity of both innate and adaptive immune system responses [7,16].

Our results are in line with other findings from the literature. In particular, in 2018, a meta-analysis [17] of 53 randomised controlled trials involving 4851 paediatric patients showed that OM-85 treatment was positively correlated with a reduction in the frequency of RTIs by more than two-fold (MD = −2.33). Moreover, the antibiotic therapy in the group receiving OM-85 was reduced by four days (MD = −4.10 days), and the duration of infection was reduced by three days with respect to the control group (MD = −3.13 days). However, the results should be interpreted with caution because of the low evidence level due to the low methodological quality and the high statistical heterogeneity among individual studies (I2 ranging from 77% to 98%). 

Another recent study conducted in a hospital centre in northern Italy between October 2016 and March 2017 found that prevalence rates of RTIs and antibiotic prescriptions in children treated for at least three months with OM-85 were almost half of the prevalence rates of RTIs and antibiotic prescriptions in children in the placebo group [18]. Authors in this study pointed out that the reduction of antibiotic overuse is a relevant additional benefit of OM-85. 

The same observations can be extended to our study. Indeed, even if the rate of co-amoxiclav prescriptions, which is considered a broad-spectrum treatment, remained the highest among all antibiotic classes, the overall number of prescriptions per child decreased significantly. 

High broad-spectrum antibiotic prescription rates were previously observed in other studies assessing common RTI treatments in both primary [19,20,21] and hospital settings [19,22] in Italy. A study assessing the appropriateness of antibiotic prescriptions in the outpatient setting in the US [23] has estimated that 30% of the prescriptions are unnecessary, mainly because they are prescribed for nonbacterial respiratory conditions. Reducing broad-spectrum antibiotic use and overall antibiotic use represents the first step in fighting the increase in antibiotic resistance.

Our study has strengths and limitations. A strength of our study lies in the utilisation of the Pedianet database that captures individual patient data from routine activities of family paediatricians. In fact, we were able to limit the misdetection of cases by assessing clinical diaries and by revising diagnoses performed in the emergency room setting. 

A limitation of our study lies in its retrospective nature. It should not be excluded that at least a few RTI cases were not seen by the FP because of the self-limiting condition or because the children were evaluated in an emergency room setting. However, this would very likely have been identified because of the need for a working parent to have a FP certification attesting the need for childcare, and hence justifying absences from work, and because the follow-up examination by the family paediatrician after the emergency room visits is always recommended after discharge, especially for younger children. Second, we did not evaluate the vaccination status (i.e., pneumococcal vaccine, Haemophilus influenzae-b vaccine) of children, which might also play a role in reducing the frequency of RTIs. Another limitation consists of a possible residual confounding due to missing data on socio-demographic factors influencing the occurrence of RTIs, such as nursery school attendance, having caregivers who smoke, and breastfeeding status. On the other hand, we analysed the same cohort of patients before and after the OM-85 prescription, thus the same cohort acted as a control of itself. Moreover, we used a proxy of 30 days to define an episode timing. In some cases (i.e., lower RTI), the healing could be slower; however, this represents an exception. Finally, we were not able to assess the usability of OM-85 since the recommended administration scheme is ten days of therapy in three consecutive months, and the package contains thirty doses in total, thus we cannot exclude administration errors in the therapy scheme. Moreover, antibiotic dosage for oral liquid formulations is based on a child′s body weight, and older children might require two packages to finish the antibiotic treatment course instead of one. 

## 5. Conclusions

We observed a significant decreasing trend of prescriptions of antibiotics and RTIs following the utilisation of OM-85. An even more marked decreasing trend was observed among males and children with recurrent RTIs.

## Figures and Tables

**Table 1:** Demographic and clinical characteristics of patients. Pedianet 2007–2017, N = 1091.

**Age, Mean (SD)**		4.3	(2.3)
**Sex**			
	Female	512	(46.93)
	Male	579	(53.07)
**Residence**			
	Nord	824	(75.53)
	Centre	102	(9.35)
	South with Islands	165	(15.13)
**Recurrent RTIs**			
	No	709	(64.99)
	Yes	382	(35.01)
Comorbidities			
	Cardiovascular problems	7	(22.58)
	Asthma	7	(22.58)
	Facial malformation	4	(12.90)
	Premature or ex-premature	3	(9.68)
	Celiac disease	3	(9.68)
	Hashimoto′s Thyroiditis-Chronic Lymphocytic Thyroiditis	3	(9.68)
	Other malformation	2	(6.45)
	Diabetes mellitus	1	(3.23)
	Down’s syndrome	1	(3.23)

**Table 2:** Antibiotic prescriptions’ frequency in 1091 children included in the PRE and POST period. Pedianet, 2007–2017.

	PRE		POST		*p*-Value *
No. of Antibiotic Prescriptions	N	(%)	N	(%)	
Mean (SD)	2.8	(2.7)	2.2	(2.6)	<0.0001
0	214	(19.62)	284	(26.03)	
1	192	(17.6)	271	(24.84)	
2	191	(17.51)	188	(17.23)	
3	145	(13.29)	123	(11.27)	
4	119	(10.91)	81	(7.42)	
≥5	230	(21.05)	144	(13.17)	

* Post-hoc test.

**Table 3:** Antibiotic prescriptions pattern in 1091 children included in the PRE and POST periods. Pedianet, 2007–2017.

Antibiotic Class	PRE		POST		
	N	(%)	N	(%)	*p*-Value *
Co-amoxiclav	888	(28.95)	677	(28.35)	0.2042
Amoxicillin	830	(27.06)	590	(24.71)	
Macrolides	584	(19.04)	441	(18.48)	
III gen. cephalosporins	428	(13.95)	376	(15.76)	
II gen. cephalosporins	197	(6.42)	178	(7.45)	
Clofoctol	27	(0.88)	26	(1.09)	
Tiamphenicole	60	(1.96)	53	(2.22)	
Others	69	(2.24)	47	(1.97)	
Total	3083		2388		

* Chi-square test.

**Table 4:** RTI frequency in 1091 children included in the PRE and POST period. Pedianet, 2007–2017.

	PRE		POST		*p*-Value *
No. of RTIs	N	(%)	N	(%)	
Mean (SD)	3.44	(2.92)	2.53	(2.56)	<0.0001
0	135	(12.37)	214	(19.62)	
1	182	(16.68)	254	(23.28)	
2	154	(14.12)	192	(17.6)	
3	189	(17.32)	151	(13.84)	
4	122	(11.18)	107	(9.81)	
≥5	309	(28.33)	126	(11.55)	

* Post-hoc test.

## Data Availability

The dataset generated during and/or analysed during the current study is available from the corresponding author upon reasonable request.

## Supplementary Materials

The following are available online at https://www.mdpi.com/article/10.3390/ijerph18136871/s1. Table S1. ICD9-CM diagnosis codes used for classification. Table S2. OM-85 prescription patterns in 1091 children included. Pedianet 2007–2017. Table S3. Antibiotic prescriptions frequency in 221 children with recurrent RTI included in the PRE and POST period. Pedianet, 2007–2017. Table S4. Antibiotic prescriptions pattern in 221 children with recurrent RTI included in the PRE and POST period. Pedianet, 2007–2017. Table S5. RTI frequency in 221 children with recurrent RTI included in the PRE and POST period. Pedianet, 2007–2017. Table S6. Antibiotic prescriptions frequency in the PRE and POST period by sex. Pedianet, 2007–2017. Table S7. RTI frequency in the PRE and POST period by sex. Pedianet, 2007–2017.

## Abbreviations

ATC	Anatomical Therapeutic Chemical
FP	Family paediatrician
ICD-9 CM	9th International Statistical Classification of Diseases and Related Health Problems system
RTI	Respiratory tract infection
SD	Standard deviation

## References

[1] Toivonen L., Karppinen S., Schuez-Havupalo L., Teros-Jaakkola T., Vuononvirta J., Mertsola J., He Q., Waris M., Peltola V. (2016). Burden of Recurrent Respiratory Tract Infections in Children: A Prospective Cohort Study. Pediatr. Infect. Dis. J..

[2] Schaad U.B., Mütterlein R., Goffin H. (2002). Immunostimulation with OM-85 in Children with Recurrent Infections of the Upper Respiratory Tract. Chest.

[3] Gutiérrez-Tarango M.D., Berber A. (2001). Safety and Efficacy of Two Courses of OM-85 BV in the Prevention of Respiratory Tract Infections in Children During 12 Months. Chest.

[4] De Benedetto F., Sevieri G. (2013). Prevention of respiratory tract infections with bacterial lysate OM-85 bronchomunal in children and adults: A state of the art. Multidiscip. Respir. Med..

[5] Cardinale F., Lombardi E., Rossi O., Bagnasco D., Bellocchi A., Menzella F. (2020). Epithelial dysfunction, respiratory infections and asthma: The importance of immunomodulation. A focus on OM-85. Expert Rev. Respir. Med..

[6] Huber M., Mossmann H., Bessler W.G. (2005). Th1-orientated immunological properties of the bacterial extract OM-85-BV. Eur. J. Med. Res..

[7] Dang A.T., Pasquali C., Ludigs K., Guarda G. (2017). OM-85 is an immunomodulator of interferon-β production and inflammasome activity. Sci. Rep..

[8] Kim E.Y., Battaile J.T., Patel A., You Y., Agapov E., Grayson M.H., A Benoit L., Byers D., Alevy Y., Tucker J. (2008). Persistent activation of an innate immune response translates respiratory viral infection into chronic lung disease. Nat. Med..

[9] Italian Marketing Authorization Authority (AIFA). https://farmaci.agenziafarmaco.gov.it/aifa/servlet/PdfDownloadServlet?pdfFileName=footer_000219_036403_RCP.pdf&retry=0&sys=m0b1l3.

[10] Esposito S., Marchisio P.G., Prada E., Daleno C., Porretti L., Carsetti R., Bosco A., Ierardi V., Scala A., Principi N. (2014). Impact of a mixed bacterial lysate (OM-85 BV) on the immunogenicity, safety and tolerability of inactivated influenza vaccine in children with recurrent respiratory tract infection. Vaccine.

[11] MiceliSopo S., Onesimo R., Giorgio V., Fundarò C., Tabacco F., Calvani M. (2011). Efficacy of over-the-counter immunostimulants in the prevention of paediatric recurrent acute respiratory tract infections. Criticisms and pitfalls of available metanalyses. Eur. Ann. Allergy. ClinImmunol..

[12] Souza F.C.D., Mocellin M., Ongaratto R., Leitão L.A.D.A., Friedrich F.O., Silveira V.D.A., Scotta M.C., Pitrez P.M., Pinto L.A. (2020). OM-85 BV for primary prevention of recurrent airway infections: A pilot randomized, double-blind, placebo-controlled study. Einstein (Sao Paulo).

[13] Carvajal J.J., Avellaneda A.M., Salazar-Ardiles C., Maya J.E., Kalergis A.M., Lay M.K. (2019). Host Components Contributing to Respiratory Syncytial Virus Pathogenesis. Front. Immunol..

[14] Upham J.W., Zhang G., Rate A., Yerkovich S.T., Kusel M., Sly P.D., Holt P.G. (2009). Plasmacytoid dendritic cells during infancy are inversely associated with childhood respiratory tract infections and wheezing. J. Allergy Clin. Immunol..

[15] Eşki A., Öztürk G.K., Gülen F., Çiçek C., Demir E. (2019). Risk Factors for Influenza Virus Related Severe Lower Respiratory Tract Infection in Children. Pediatr. Infect. Dis. J..

[16] Rossi G.A., Bessler W., Ballarini S., Pasquali C. (2018). Evidence that a primary anti-viral stimulation of the immune response by OM-85 reduces susceptibility to a secondary respiratory bacterial infection in mice. Ital. J. Pediatr..

[17] Yin J., Xu B., Zeng X., Shen K. (2018). Broncho-Vaxom in pediatric recurrent respiratory tract infections: A systematic review and meta-analysis. Int. Immunopharmacol..

[18] Esposito S., Bianchini S., Bosis S., Tagliabue C., Coro I., Argentiero A., Principi N. (2019). A randomized, placebo-controlled, double-blinded, single-centre, phase IV trial to assess the efficacy and safety of OM-85 in children suffering from recurrent respiratory tract infections. J. Transl. Med..

[19] Messina F., Clavenna A., Cartabia M., Piovani D., Bortolotti A., Fortino I., Merlino L., Bonati M. (2019). Antibiotic prescription in the outpatient paediatric population attending emergency departments in Lombardy, Italy: A retrospective database review. BMJ Paediatr. Open.

[20] Costenaro P., Cantarutti A., Barbieri E., Scamarcia A., Oletto A., Sacerdoti P., Lundin R., Cantarutti L., Giaquinto C., Donà D. (2021). Antibiotic Prescriptions for Children With Community-acquired Pneumonia: Findings From Italy. Pediatr. Infect. Dis. J..

[21] Barbieri E., Donà D., Cantarutti A., Lundin R., Scamarcia A., Corrao G., Cantarutti L., Giaquinto C. (2019). Antibiotic prescriptions in acute otitis media and pharyngitis in Italian pediatric outpatients. Ital. J. Pediatr..

[22] Barbieri E., De Luca M., Minute M., D’Amore C., Degli Atti M.L.C., Martelossi S., Giaquinto C., Da Dalt L., Zaoutis T., Dona D. (2020). Impact and Sustainability of Antibiotic Stewardship in Pediatric Emergency Departments: Why Persistence Is the Key to Success. Antibiotics.

[23] Fleming-Dutra K.E., Hersh A.L., Shapiro D.J., Bartoces M., Enns E., File T.M., Finkelstein J.A., Gerber J.S., Hyun D.Y., Linder J.A. (2016). Prevalence of Inappropriate Antibiotic Prescriptions Among US Ambulatory Care Visits, 2010–2011. JAMA.

