# Supplementary material for: Use of the Bacterial Lysate OM-85 in the Paediatric Population in Italy: A Retrospective Cohort Study
Source: Int J Environ Res Public Health. 2021 Jun 26;18(13):6871. doi: 10.3390/ijerph18136871 (PMC8297025; doi:10.3390/ijerph18136871)
Supplement: Supplementary file 1 [file ijerph-18-06871-s001.zip › ijerph-1238312-SI.pdf]

Supplementary material

Tables

Table S1. ICD9-CM diagnosis codes used for classification.

Table S2. OM-85 prescription patterns in 1091 children included. Pedianet 2007-2017.

Table S3. Antibiotic prescriptions frequency in 221 children with recurrent RTI included in the PRE and POST period. Pedianet, 2007-2017.

Table S4. Antibiotic prescriptions pattern in 221 children with recurrent RTI included in the PRE and POST period. Pedianet, 2007-2017.

Table S5. RTI frequency in 221 children with recurrent RTI included in the PRE and POST period. Pedianet, 2007-2017.

Table S6. Antibiotic prescriptions frequency in the PRE and POST period by sex. Pedianet, 2007-2017.

Table S7. RTI frequency in the PRE and POST period by sex. Pedianet, 2007-2017.

Table S1. ICD9-CM diagnosis codes used for classification.

Diagnosis	ICD9-CM code
**Pneumonia**	481 Pneumococcal pneumonia [Streptococcus pneumoniae pneumonia],<br>482 Other bacterial pneumonia<br>483 Pneumonia due to other specified organism<br>484 Pneumonia in infectious diseases classified elsewhere,<br>485 Bronchopneumonia, organism unspecified,<br>486 Pneumonia, organism unspecified
**Pharyngitis**	462 Acute pharyngitis<br>463 Acute tonsillitis,<br>034 Streptococcal sore throat and scarlet fever
**Sinusitis**	461 Acute sinusitis<br>473 Chronic sinusitis
**Suppurative otitis media**	382 Suppurative and unspecified otitis media
**Non-suppurative otitis media**	381: Non-suppurative otitis media and Eustachian tube disorders
**Upper respiratory tract infection**	464 Laryngitis- tracheitis<br>460.x Upper respiratory tract infection
**Lower respiratory tract infection**	490 Acute bronchitis<br>466 Bronchiolitis<br>491Chronic bronchitis

Table S2. OM-85 prescription patterns in 1091 children included. Pedianet 2007-2017.

**# of OM-85 prescriptions**		**N**	**(%)**
	1	876	80.29
	2	159	14.57
	3	36	3.3
	≥4	20	1.83

Table S3. Antibiotic prescriptions frequency in 221 children with recurrent RTI included in the PRE and POST period. Pedianet, 2007-2017.

**# of antibiotic prescriptions**	**PRE** N	(%)	**POST** N	(%)	p-value*
Mean (SD)	4.1	(1.3)	2.9	(1.7)	< 0.0001
0	5	(2.4)	24	(11.4)	
1	8	(3.8)	33	(15.6)	
2	14	(6.6)	34	(16.1)	
3	33	(15.6)	34	(16.1)	
4	25	(11.9)	29	(13.7)	
≥ 5	126	(59.7)	57	(27)	

* Post-hoc test

Table S4. Antibiotic prescriptions pattern in 221 children with recurrent RTI included in the PRE and POST period. Pedianet, 2007-2017.

**Antibiotic class**	**PRE**		**POST**		**p-value***
	**N**	**(%)**	**N**	**(%)**	
Co-amoxiclav	315	(27.02)	205	(26.32)	0.6134
Amoxicillin	297	(25.47)	194	(24.9)	
Macrolides	206	(17.67)	126	(16.18)	
III gen cephalosporins	186	(15.95)	131	(16.81)	
II gen cephalosporins	107	(9.18)	84	(10.79)	
Clofoctol	14	(1.2)	16	(2.05)	
Tiamphenicole	21	(1.8)	14	(1.8)	
Others	20	(1.71)	9	(1.17)	
Total	1166		779		

* Chi-square test

Table S5. RTI frequency in 221 children with recurrent RTI included in the PRE and POST period. Pedianet, 2007-2017.

**# of RTI**	**PRE**		**POST**		**p-value***
	**N**	**%**	**N**	**%**	
Mean (std)	8.17	(2.47)	4.48	(3.39)	< 0.0001
≤5	-	-	153	(72.51)	
6	59	(27.96)	16	(7.58)	
7	51	(24.17)	7	(3.32)	
8	34	(16.11)	10	(4.74)	
≥9	67	(31.76)	25	(11.84)	

* Post-hot test

Table S6. Antibiotic prescriptions frequency in the PRE and POST period by sex. Pedianet, 2007-2017.

	Male				Female			
Mean (std)	2.5	(1.8)	1.9	(1.7)	2.3	(1.8)	1.8	(1.7)
0	109	(18.8)	141	(24.4)	105	(20.5)	143	(27.9)
1	101	(17.4)	142	(24.5)	91	(17.8)	129	(25.2)
2	94	(16.2)	106	(18.3)	97	(19)	82	(16)
3	75	(13)	72	(12.4)	70	(13.7)	51	(10)
4	73	(12.6)	44	(7.6)	46	(9)	37	(7.2)
≥5	127	(21.9)	74	(12.8)	103	(20.1)	70	(13.7)
p-value	< 0.0001				< 0.0001			

Table S7. RTI frequency in the PRE and POST period by sex. Pedianet, 2007-2017.

	Male				Female			
Mean (std)	2.9	(1.8)	2.3	(1.7)	2.7	(1.7)	2.1	(1.7)
0	70	(12.1)	107	(18.5)	65	(12.7)	107	(20.9)
1	93	(16.1)	127	(21.9)	89	(17.4)	127	(24.8)
2	70	(12.1)	105	(18.1)	84	(16.4)	87	(17)
3	104	(18)	82	(14.2)	85	(16.6)	69	(13.5)
4	60	(10.4)	60	(10.4)	62	(12.1)	47	(9.2)
≥5	182	(31.4)	98	(16.9)	127	(24.8)	75	(14.7)
p-value	< 0.0001				< 0.0001			
